# The effect of respiratory viral assay panel on antibiotic prescription patterns at discharge in adults admitted with mild to moderate acute exacerbation of COPD: a retrospective before- after study

**DOI:** 10.1186/s12890-019-0872-0

**Published:** 2019-07-01

**Authors:** Mayanka Tickoo, Robin Ruthazer, Amit Bardia, Shira Doron, Gabriela M. Andujar-Vazquez, Bradley J. Gardiner, David R. Snydman, Sebastian G. Kurz

**Affiliations:** 1 Department of Internal Medicine, Division of Pulmonary, Sleep and Critical Care, Yale School of Medicine, New Haven, CT USA; 2 Biostatistics, Epidemiology, Research, and Design Center, Tufts Clinical and Translational Science Institute, Tufts University School of Medicine, Boston, MA USA; 3 Department of Anesthesiology, Yale School of Medicine, New Haven, CT USA; 4 Division of Geographic Medicine and Infectious Diseases, Tufts Medical Center, Tufts University School of Medicine, Boston, MA USA; 5 Pulmonary and Critical Care Division, Mount Sinai National Jewish Respiratory Institute, New York, NY USA

**Keywords:** COPD, Viral assay panel, Antibiotic prescription

## Abstract

**Background:**

Despite well-defined criteria for use of antibiotics in patients presenting with mild to moderate Acute Exacerbation of Chronic Obstructive Pulmonary Disease (AECOPD), their overuse is widespread. We hypothesized that following implementation of a molecular multiplex respiratory viral panel (RVP), AECOPD patients with viral infections would be more easily identified, limiting antibiotic use in this population. The primary objective of our study was to investigate if availability of the RVP decreased antibiotic prescription at discharge among patients with AECOPD.

**Methods:**

This is a single center, retrospective, before (pre-RVP) - after (post-RVP) study of patients admitted to a tertiary medical center from January 2013 to March 2016. The primary outcome was antibiotic prescription at discharge. Groups were compared using univariable and multivariable logistic-regression.

**Results:**

A total of 232 patient-episodes were identified, 133 following RVP introduction. Mean age was 68.1 (pre-RVP) and 68.3 (post-RVP) years respectively (*p* = 0.88). Patients in pre-RVP group were similar to the post-RVP group with respect to gender (*p* = 0.54), proportion of patients with BMI < 21(*p* = 0.23), positive smoking status (*p* = 0.19) and diagnoses of obstructive sleep apnea (OSA, *p* = 0.16). We found a significant reduction in antibiotic prescription rate at discharge in patients admitted with AECOPD after introduction of the respiratory viral assay (pre-RVP 77.8% vs. post-RVP 63.2%, *p* = 0.01). In adjusted analyses, patients in the pre-RVP group [OR 2.11 (CI: 1.13–3.96), *p* = 0.019] with positive gram stain in sputum [OR 4.02 (CI: 1.61–10.06), *p* = 0.003] had the highest odds of antibiotic prescription at discharge.

**Conclusions:**

In patients presenting with mild to moderate Acute Exacerbation of Chronic Obstructive Pulmonary Disease (AECOPD), utilization of a comprehensive respiratory viral panel can significantly decrease the rate of antibiotic prescription at discharge.

## Background

The global burden of chronic obstructive pulmonary disease (COPD) is extremely high, with an annual predicted mortality of 3 million in 2015 [1]. In the United States alone, an estimated 16 million people suffer from COPD [2] with a direct estimated annual health care cost of $32 billion [3]. Acute exacerbations of COPD (AECOPD) are a frequent precipitant for hospitalization, and about 70% of cases can be attributed to respiratory infections [4]. Of these, a viral etiology is estimated in 30–50% of cases [5] [6]. While for many healthy individuals, respiratory viral illnesses (RVIs) are acute and self-limiting, patients with COPD are particularly vulnerable to prolonged and complicated clinical courses [7]. Currently, treatment with antibiotics is recommended in certain patient populations with AECOPD such as those who are critically ill or mechanically ventilated [8]. However, the routine use of antibiotics has shown inconsistent benefits in patients who do not require ICU admission [9]. There is an increasing pressure on our hospital systems to diagnose, treat and discharge patients quickly, which has led to overuse of antibiotics even for viral etiologies of various infections. However, given the dwindling pipeline of new antimicrobials as well as the rising burden of antimicrobial resistance, judicious use of antibiotics by limiting their use to patients who actually have a bacterial infection is of paramount importance [10].

Distinguishing viral infections from other causes of AECOPD has historically been difficult, resulting in diagnostic uncertainty, overuse of antibiotics, and prolonged hospitalization. Over the last 10–15 years there has been a rapid increase in the availability of newer molecular diagnostics for respiratory viruses, which are more accurate, faster and easier to use than older methods. While molecular assays are being integrated into clinical diagnostics at an impressive rate, their effect on medical decision-making at the bedside is not well described.

We hypothesized that by integration of fast, highly sensitive respiratory viral panels (RVP) into clinical care, AECOPD patients with viral infection as their primary trigger can be more easily identified, allowing health care providers to feel more comfortable limiting antibiotic use resulting in an overall decrease in antibiotic prescription at discharge. We therefore conducted a retrospective analysis of data from our center and assessed the pattern of antibiotic prescription at discharge before and after the introduction of a molecular multiplex viral assay in patients admitted to medical floors with acute exacerbations of COPD.

## Methods

This was a single center, retrospective, before (pre-RVP) - after (post-RVP) study to assess the difference in antibiotic prescription at discharge following availability of a PCR based, rapid, multiplex diagnostic viral assay as an intervention in adult patients admitted to the hospital floor with acute exacerbation of COPD. Sample size calculations were performed a priori indicating we would need 99 patients in each group to have 80% power to detect a > =20% difference between groups.

After obtaining Institutional Board Review (IRB) approval, medical records of patients admitted to a tertiary care hospital from January 2013 to March 2016 were reviewed. All adult patients with a primary billing code of 491.21 (obstructive chronic bronchitis with exacerbation) in ICD-9 and J44.1 (chronic obstructive pulmonary disease with exacerbation) in ICD-10 admitted to non-ICU level hospital care were included in the study. Exclusion criteria included patients with concomitant diagnoses of pneumonia, chest X ray reports reading ‘infiltrates’, sputum cultures growing typical or atypical bacterial organisms, coexisting other infections (UTI, bacteremia) that would necessitate antibiotic therapy, readmission within 6 weeks of a prior admission for AECOPD and unplanned discharge such as in-hospital mortality, or patients who left against medical advice. Patients with severe respiratory failure requiring admission to the intensive care unit in the setting of acute exacerbation of COPD were also excluded from the study. Of note, patients with positive result on Gram Stain but negative cultures were not excluded from the study. Each admission was identified as a single patient-care episode.

### Covariates

All the covariates were decided a priori which included age, gender, BMI < 21, smoking status (never smoker, former smoker, current smoker), comorbidities including obstructive sleep apnea, coronary artery disease, congestive heart failure, atrial fibrillation, diabetes, hypertension and chronic kidney disease (stage II or higher), use of home oxygen prior to admission, use of chronic steroids for COPD prior to admission, sub-specialty service on admission (i.e. inpatient pulmonary, inpatient infectious diseases, others), as well as use of high flow nasal oxygen or non-invasive ventilation during the admission. BMI was dichotomized as below and above 21 because patients with COPD with BMI under 21 have clinically been shown to have poor 5 year survival [11]. We compared the rate of antibiotic prescription at discharge before and after the availability of the PCR based Respiratory Viral Panel (August 2014).

### Assay

The Biofire FilmArray® Respiratory Viral Panel (BioFire Diagnostics, Salt Lake City, a bioMérieux company, Marcy, l’Etoile, France) is a comprehensive FDA approved panel for detecting 20 respiratory viruses including influenza A and B, parainfluenza 1–4, human metapneumovirus, human rhinovirus/enterovirus, 4 coronaviruses, adenovirus, respiratory syncytial virus (RSV) as well as 3 bacteria (*Bordetella pertussis, Chlamydophila pneumoniae, Mycoplasma pneumoniae*). It involves nested multiplex PCR of study samples and utilizes endpoint melting curve data automatically generating a targeted report of individual viral targets. Its clinical use and validation has been previously described, and it was performed according to the manufacturers instructions without protocol variations [12].

### Outcome

The primary outcome of the study was rate of prescription of any antibiotic at discharge for patients admitted to the medical floor with acute exacerbation of COPD before (pre-RVP) and after (post-RVP) routine availability of the RVP.

### Statistical analysis

All categorical variables were described using frequencies and proportions. Means and standard deviations (SD) were used for continuously coded variables. Chi-square and Student’s t-test or Kruskal-Wallis test were used to compare proportions and means or medians respectively. Patients were stratified according to pre vs. post RVP groups and proportion of patients with antibiotics prescribed at discharge were compared between the two groups. We used logistic regression to estimate unadjusted and adjusted odds ratios for the association of post vs. pre RVP with the outcome of antibiotic use. The adjusted model included home O2 and chronic steroids as a priori determined factors to include as covariates together with any study variables that had unadjusted *p*-values <.20 for the association with antibiotic use. Microsoft excel, SPSS and SAS programs were used for data storage and analysis.

## Results

Baseline characteristics stratified by testing period (pre-RVP and post-RVP) are listed in Table 1. A total of 232 patient-episodes were identified of which 133 occurred after RVP introduction. The mean age of patients in the two groups was 68.1 (pre-RVP) and 68.3 (post-RVP) years respectively (*p* = 0.88). There was no significant difference between the groups with respect to gender, proportion of patients with BMI < 21, smoking status, or comorbidities including obstructive sleep apnea, congestive heart failure and chronic kidney disease (CKD, stage 2 or higher). Compared to the pre-RVP group, patients in the post-RVP group had a higher rate of hypertension (51.5% vs. 64.7%, *p* = 0.04), Diabetes mellitus (19.2% vs. 34.6%, *p* = 0.009), and coronary artery disease (21.2% vs. 35.3%, *p* = 0.019), as well as lower rates of chronic steroid use (17.2% vs. 6.8%,*p* = .001). There were no differences between the two groups with respect to use of non-invasive ventilation or high flow nasal oxygen during admission (*p* = 0.36), positive sputum Gram stain(*p* = 0.38), or medical sub-specialty service at discharge [Pulmonary vs. Infectious disease vs. others] (*p* = 0.26).

**Table 1:** Demographics and clinical variables in 232 patients admitted to medical floors at tertiary medical center with acute exacerbation of COPD; January 2013 – March 2016

BASELINE CHARACTERISTICS	Total (*n* = 232)	Pre-RVP (*n* = 99)	Post-RVP (*n* = 133)	*p*
Age years; mean (SD)	68.3 (11.6)	68.1 (11.8)	68.3 (11.5)	0.8844
Females; n (%)	122 (52.8)	50 (50.9)	72 (54.5)	0.5427
BMI > 21; n (%)	182 (78.4)	74 (74.7)	108 (81.2)	0.2369
Smoking status				
Current smokers; n (%)	92 (39.7)	44 (44.4)	48 (36.1)	0.1982
Ever smokers; n (%)	203 (87.5)	85 (85.9)	118 (88.7)	0.5143
Comorbidities				
Obstructive Sleep Apnea; n (%)	37 (15.9)	12 (12.1)	25 (18.8)	0.1696
Hypertension; n (%)	137 (59.1)	51 (51.5)	86 (64.7)	**0.0440**
Diabetes Mellitus; n (%)	65 (28.0)	19 (19.2)	46 (34.6)	**0.0098**
Coronary Artery Disease; n (%)	68 (29.3)	21 (21.2)	47 (35.3)	**0.0194**
CHF; n (%)	63 (27.2)	23 (23.2)	40 (30.1)	0.2464
Atrial Fibrillation; n (%)	29 (12.5)	11 (11.1)	18 (13.5)	0.5810
CKD stage 2+; n (%)	20 (8.6)	11 (11.1)	18 (13.5)	0.5810
Chronic steroid dependence; n (%)	26 (11.2)	17 (17.2)	9 (6.8)	**0.0130**
Home oxygen use; n (%)	93 (40.1)	36 (36.4)	57 (42.9)	0.3182
Positive sputum Gram stain; n (%)	50 (21.6)	24 (24.2)	26 (19.5)	0.3898
NIV/HFNC use during admission; n (%)	32 (13.8)	16 (16.2)	16 (12.0)	0.3667
Medical Service at discharge; n (%)				
Pulmonary	84 (36.2)	41 (41.4)	43 (32.3)	0.2694
Infectious Diseases	19 (8.2)	9 (9.1)	10 (7.5)	
Other	129 (55.6)	49 (49.5)	80 (60.2)	

*SD* standard deviation, *Pre RVP* pre Respiratory Viral Panel group, *Post RVP* post Respiratory Viral Panel group, *BMI* Body Mass Index

**Fig. 1:**
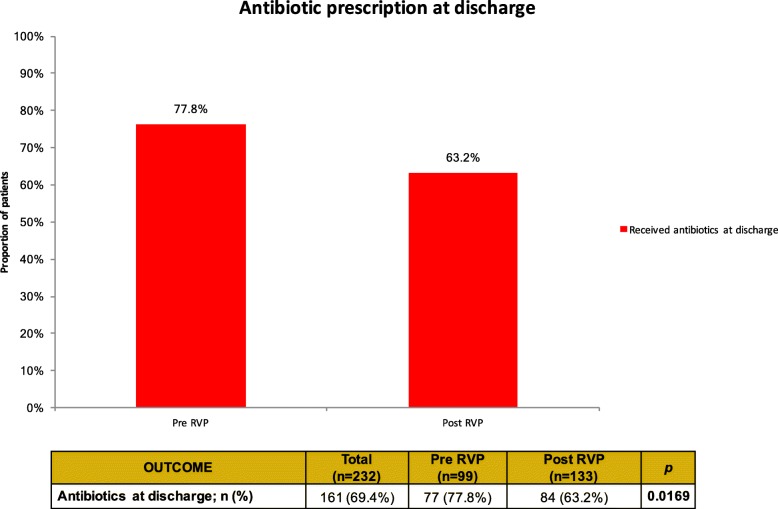
Proportion of patients receiving antibiotics at discharge comparing pre RVP and post RVP groups. *Pre RVP* pre Respiratory Viral Panel group, *Post RVP* post Respiratory Viral Panel group

Figure 1 depicts the percentage of patients who were prescribed antibiotics at discharge among the two groups. We found a significant reduction in antibiotic prescription rate at discharge in patients admitted with AECOPD after introduction of the respiratory viral assay (pre-RVP 77.8% vs. post-RVP 63.2%, p = 0.01) in the patient cohort at our center

In adjusted analyses (Table 2), patients in the pre-RVP group had higher odds of antibiotic prescription at discharge [OR 2.11 (95% CI: 1.13–3.96), p = 0.019] controlling for home O2 use, chronic steroids, BMI > 21 and positive gram stain in sputum. Presence of a positive gram stain in sputum was also found to have a significant independent association with antibiotics at discharge [OR 4.02 (CI: 1.61–10.06), *p* = 0.003]. Home oxygen use and chronic steroid use, included in the models based on a priori hypothesized association, were not statistically significantly associated with antibiotic use at discharge in our model (*p* > 0.90 for both).

**Table 2:** Multivariable Logistic Regression model for factors influencing antibiotic prescription at discharge in patients admitted to medical floor with acute exacerbation of COPD

Factors	OR (95% CI)	*p* value
Lack of RVP (pre RVP)	2.11 (CI: 1.13–3.96)	**0.0192**
Home oxygen use	1.03 (CI: 0.56–1.89)	0.9312
Chronic steroid dependence	1.04 (CI: 0.39–2.76)	0.9436
BMI > 21	1.89 (CI: 0.95–3.76)	0.0694
Positive sputum Gram stain	4.02 (CI: 1.61–10.06)	**0.0029**

*OR* odds ratio, *CI* confidence interval, *RVP* respiratory viral panel

## Discussion

Our results show a significant decline in the rate of antibiotic prescription at discharge following routine RVP availability in patients admitted to medical floors with acute COPD exacerbations. Aside from availability of the multiplex viral detection panel, presence of positive sputum gram stain was the only other significant factor found to be associated with antibiotic prescription at discharge. Approximately one third of patients in our study had a positive RVP result which is comparable to other studies [13, 14].

The results of the study should be interpreted with caution. Viral respiratory infections may increase the risk of bacterial co-infections in COPD patients. Also, a positive viral respiratory panel does not rule out a coexisting bacterial infection. Our study suggests that in carefully selected patients (mild -moderate COPD exacerbations, negative bacterial sputum cultures and no infiltrates on chest X ray (exclusion criteria), the availability of a viral assay may change antibiotic prescription pattern at discharge.

Several approaches have been tried to identify patients with acute exacerbations of COPD who are most likely to benefit from antibiotic therapy. While antibiotic use in patients with severe COPD exacerbations requiring ICU level care is recommended, there are no clear guidelines addressing those with less severe infections [15]. It is well known that in patients with bacterial exacerbations of COPD, antibiotics reduce illness severity acutely as well as the risk of subsequent exacerbations and mortality [16]. Classically, Anthonisen et al. have formulated clinical criteria to identify bacterial infections [17]. Based on these criteria, guidelines from the Global Initiative for Chronic Obstructive Lung Disease (GOLD) advocate for use of antibiotics in patients who present with increasing dyspnea, sputum purulence and sputum volume [18], as well as patients with two of the above cardinal symptoms who require invasive or non-invasive mechanical ventilation [18]. Similarly, the Canadian Thoracic Society guidelines recommend antibiotics in more severe purulent AECOPD (new increased expectoration of mucopurulent sputum and dyspnea) [19] in the outpatient setting. Not all patients however, necessarily present with increased purulent sputum production or have a positive gram stain in acute COPD exacerbations, and it is well known that in current practice, antibiotics are often overprescribed irrespective of clinical criteria [16]. Given the dwindling pipeline of antibiotics and the rising antimicrobial resistance, there is a push to identify biochemical or microbiologic markers to identify non-bacterial episodes of AECOPD to try and limit antibiotic use to appropriate clinical scenarios.

Historic viral diagnostic techniques such as serology, culture, enzyme immunoassay, and immunofluorescence were limited by poor sensitivity, complex methodology and slow turn-around time which impeded their inclusion in diagnostic algorithms thus far. The development of molecular multiplex assays for the rapid and accurate detection of respiratory viruses over the last decade has been a major step forward and has led to a greater appreciation of their ubiquity and contribution to many disease states [20–22]. Multiplex assays also allow for simultaneous detection of multiple pathogens [23] with a high degree of sensitivity and specificity [12]. The impact of viral testing on antibiotic prescription has shown variable results in the literature. In children hospitalized with bronchiolitis, testing for viral pathogens did not show an impact on use of antibiotics or other care parameters including length of stay or need for oxygen [24, 25]. In another study analyzing use of a multiplex assay for 9 viruses, no significant decrease was noted in antibiotic prescription in all adult patients admitted with respiratory symptoms of fever, cough and shortness of breath [26]. Similarly, Hernes et.al. in a general hospital setting with elderly patient noted little impact on the antimicrobial treatment or length of hospitalization based on access to early viral diagnosis by real-time PCR [27]. However, these studies utilized single pathogen isolation techniques or used multiplex assays that could detect fewer [9–14] pathogens. More recently, in a study on pediatric patients hospitalized with acute respiratory illness (ARI), McCollough et.al. showed that RVP testing may enhance physician decision-making when prescribing antimicrobials in children hospitalized with acute respiratory infections [28]. In an outpatient setting in adults, Green et.al. evaluated the role of the multiplex respiratory viral assay in adult patients with COPD exacerbation and found that testing positive for influenza virus was associated with receiving fewer antibiotic prescriptions [14] at their center, however, there was no difference in antibiotic prescription if patients had non-influenza viruses on the multiplex assay. Our study adds to this body of literature, distilling the patient population down to adult patients with mild to moderate COPD exacerbation who need hospitalization for bronchodilators and or steroids, but might not need to be treated with antibiotics. It is conceivable that the rapid identification of respiratory viruses in real-time has an impact on clinical decision-making, providing clinicians with the confidence to discontinue antibiotics for non-bacterial exacerbations of COPD.

Our study highlights the issue of judicious antibiotic prescription practices for COPD exacerbations. As such, up to 30% of all exacerbations are triggered by enhanced eosinophilic inflammation in the airways. While the non-infectious causes of exacerbation of COPD will not be helped by use of viral panels, these panels may further decrease antibiotic prescription in patients with an infectious etiology for COPD exacerbation.

Our study has certain limitations. It is a single center, retrospective study which has its inherent shortcomings. The data was collected cumulatively over a 38-month period, without assessing for seasonal variation in viral influenza like illnesses (ILIs) that are known to associated with incidence of acute COPD exacerbations. We also did not adjust the data for reported prevalence or severity of various ILIs between 2013 and 2016. However, since the goal of the study was to evaluate the antibiotic prescription pattern at discharge specific to each patient contact, we do not believe that variation in prevalence of respiratory infections would affect the rate of antibiotic prescription. Our study spanned the conversion from ICD 9 to ICD 10 coding systems, which, despite being agreed upon as transferable in terms of primary diagnostics, could miss or misclassify certain patients during our study period. Our study was not designed to assess re-admission rates and adverse outcomes of a RVP based antibiotic prescription approach. In concordance with good clinical judgement, we have excluded patients with radiographic evidence of pneumonia, given that most providers would feel uncomfortable attributing a radiographic infiltrate to viral pneumonia only, rather than suspecting and treating for bacterial co- / super infection in a patient with viral respiratory illness. Also, we were not able to assess the role of procalcitonin guided prescription practices in our patient population as procalcitonin levels were not routinely measured in our hospital in the study period. Procalcitonin use has been shown to safely reduce antibiotic prescription [29]. Further studies are required to assess if RVP has an addition impact in prescription practices where procalcitonin based antibiotic prescription is practiced. Our cohort did not include outpatient visits. Mild to moderate COPD exacerbation included patients who needed hospitalization but did not need ICU admission. Finally, the RVP is an expensive test and formal cost-effectiveness analyses would be required to determine its value in this setting.

## Conclusion

In patients presenting with Acute Exacerbation of Chronic Obstructive Pulmonary Disease (AECOPD), utilization of a comprehensive RVP can significantly decrease the rate of antibiotic prescription at discharge. We believe that widespread use of diagnostic viral panels will allow for more targeted use of antibiotics in patients with mild to moderate viral COPD exacerbations who do not meet Antonisen’s criteria, thereby reducing antibiotic misuse and help combat the threat of antimicrobial resistance. Further studies are required to examine the effect of this practice on patient outcomes and antibiotic resistance patterns in the community.

## Data Availability

The data is not publicly available database. Our current IRB does not allow us to share data publicly.

## Abbreviations

AECOPD: Acute Exacerbation of Chronic Obstructive Pulmonary Disease
BMI: Body mass index
COPD: Chronic obstructive pulmonary disease
IRB: Institutional Review Board
post-RVP: After institution of respiratory viral panel
pre-RVP: Before institution of respiratory viral panel
RVI: Respiratory viral illnesses
RVP: Respiratory Viral Panel
